# Very-low-dose twice-daily aspirin maintains platelet inhibition and improves haemostasis during dual-antiplatelet therapy for acute coronary syndrome

**DOI:** 10.1080/09537104.2019.1572880

**Published:** 2019-02-13

**Authors:** William A.E. Parker, Rachel C. Orme, Jessica Hanson, Hannah M. Stokes, Claire M. Bridge, Patricia A. Shaw, Wael Sumaya, Kirstie Thorneycroft, Giovanna Petrucci, Benedetta Porro, Heather M. Judge, Ramzi A. Ajjan, Bianca Rocca, Robert F. Storey

**Affiliations:** 1 Department of Infection, Immunity and Cardiovascular Disease, University of Sheffield, Sheffield, United Kingdom; 2 Department of Cardiology, Sheffield Teaching Hospitals NHS Foundation Trust, Sheffield, UK; 3 Institute of Pharmacology, Catholic University School of Medicine, Rome, Italy; 4 Centro Cardiologico Monzino, Istituto di Ricovero e Cura a Carattere Scientifico Cardiologico Monzino, Milan, Italy; 5 Leeds Institute of Cardiovascular and Metabolic Medicine, University of Leeds, Leeds, UK

**Keywords:** Aspirin, bleeding, P2Y_12_ inhibitors, thromboxane, ticagrelor

## Abstract

Higher aspirin doses may be inferior in ticagrelor-treated acute coronary syndrome (ACS) patients and reducing bleeding risk whilst maintaining antithrombotic benefits could improve outcomes. We characterized the pharmacodynamics of a novel dual-antiplatelet-therapy regimen consisting of very-low-dose twice-daily (BD) aspirin with standard-dose ticagrelor. A total of 20 ticagrelor-treated ACS patients entered a randomized crossover to take aspirin 20 mg BD (12-hourly) during one 14-day period and 75 mg once-daily (OD) in the other. After 14 days of treatment, serum thromboxane (TX)B_2_ and light-transmittance aggregometry were assessed pre- and 2 h post-morning-dose, bleeding time was measured post-dose, and TXA_2_ and prostacyclin stable metabolites were measured in urine collected 2 h post-morning-dose. Data are expressed as mean ± SD. After 14 days treatment, serum TXB_2_ levels were significantly greater 2 h post-dosing with aspirin 20 mg BD vs. 75 mg OD (3.0 ± 3.6 ng/mL vs. 0.8 ± 1.9 ng/mL; *p* = 0.018) whereas pre-dosing levels were not significantly different (3.5 ± 4.1 ng/mL vs. 2.5 ± 3.1 ng/mL, *p* = 0.23). 1-mmol/L arachidonic acid-induced platelet aggregation was similarly inhibited by both regimens pre-dose (8.5 ± 14.3% vs. 5.1 ± 3.6%, *p* = 0.24) and post-dose (8.7 ± 14.2% vs. 6.6 ± 5.3%; *p* = 0.41). Post-dose bleeding time was shorter with 20 mg BD (680 ± 306 s vs. 834 ± 386 s, *p* = 0.02). Urinary prostacyclin and TX metabolite excretion were not significantly different. In conclusion, compared to aspirin 75 mg OD, aspirin 20 mg BD provided consistent inhibition of platelet TXA_2_ release and aggregation, and improved post-dose hemostasis, in ticagrelor-treated ACS patients. Further studies are warranted to assess whether this regimen improves the balance of clinical efficacy and safety.

## Abbreviations

8-iso-PGF_2α_: 8-iso prostaglandin 2α
ACS: acute coronary syndrome
BD: twice-daily
COX: cyclo-oxygenase
DAPT: dual antiplatelet therapy
MACE: major adverse cardiovascular events
OD: once-daily
PLATO: PLATelet inhibition and patient Outcomes
PGI_2_: prostacyclin
TX: thromboxane

## Introduction

The combination of the irreversible cyclo-oxygenase (COX) inhibitor aspirin (acetylsalicylic acid) 75–100 mg once-daily (OD) and the P2Y_12_ inhibitor ticagrelor 90 mg twice-daily (BD) represents a standard recommended regimen of dual antiplatelet therapy (DAPT) in acute coronary syndromes (ACS) [1–4]. The PLATelet inhibition and patient Outcomes (PLATO) study showed that DAPT with aspirin and ticagrelor was superior to the previous standard regimen of aspirin and clopidogrel in ACS, reducing the incidence of major adverse cardiovascular events (MACE). Despite such potent antiplatelet therapy, the residual MACE risk remains around 10% at 1 year [5].

Continuing DAPT with aspirin and reduced-dose ticagrelor long-term in stable high-risk individuals further decreases the risk of MACE compared with aspirin alone [6]. However, increasing the potency or duration of DAPT also leads to increased bleeding, which is associated not only with higher mortality but also patient distress, inconvenience, and premature discontinuation, even if events are considered minor or trivial by clinicians [7,8]. Concern about bleeding risk may, therefore, dissuade clinicians from recommending DAPT, particularly in the long-term, to the potential detriment of high-risk patients.

Further subgroup analysis of the PLATO study suggested that doses of aspirin ≥ 300 mg were associated with reduced benefit of ticagrelor over clopidogrel and, as a result, daily aspirin doses >100 mg are not recommended when used in DAPT [9,10].

Aspirin and ticagrelor have additive antithrombotic effects [11]. Whilst ticagrelor inhibits the platelet adenosine diphosphate P2Y_12_ receptor, aspirin’s action relates to its ability to irreversibly inhibit platelet COX-1, thus reducing prothrombotic eicosanoid TXA_2_ production. Aspirin can achieve almost-complete (≥95%) inhibition of TXA_2_ biosynthesis in healthy subjects at repeated daily doses as low as 20–30 mg [12–14]. At doses above 300 mg, aspirin also inhibits COX-2 in humans, a key enzyme in the pathway of constitutive, vascular-protective prostacyclin (PGI_2_) release by the endothelium [12,15]. Inhibition of COX-2 and PGI_2_ biosynthesis may be counterproductive in patients with ACS: both traditional and COX-2-selective nonsteroidal anti-inflammatory drugs are associated with an increased risk of MACE [16–18]. There is also evidence from animal studies that ticagrelor may beneficially induce COX-2 and endothelial nitric oxide synthase through an adenosine-dependent mechanism, which may limit myocardial infarct size through synergistic antiplatelet and vasodilatory effects of PGI_2_, nitric oxide and P2Y_12_ inhibition [19–21]. Therefore, inhibition of COX-2 by high-dose aspirin may compromise the beneficial effects of ticagrelor on this pathway.

Aspirin dosing frequency may also influence pharmacodynamic efficacy: OD aspirin administration may be insufficient to maintain consistency of platelet inhibition over 24 h in patients with higher platelet turnover, including smokers and those with obesity or diabetes mellitus, or in individuals undergoing procedural intervention [22–27]. Multiple daily-dosing regimens of aspirin have been shown to improve consistency of pharmacodynamic effect, not only in conditions with extreme acceleration of platelet turnover, such as essential thrombocythemia [28–30], but also in patients with ischemic heart disease [25,27,31–33] when administered as single antiplatelet therapy. However, such regimens of aspirin have not been studied in those receiving dual antiplatelet therapy (DAPT) with ticagrelor, which is already given BD, in whom the overall pharmacodynamic profile of DAPT may potentially be improved by BD aspirin dosing.

An ideal regimen of aspirin and ticagrelor would be one that maintains the anti-ischemic benefit of DAPT through effective and steady inhibition of platelet COX-1 and P2Y_12_, ensures consistency of effect across the dosing interval, avoids inhibition of vasoprotective COX-2-dependent PGI_2_ biosynthesis and has minimized effects on hemostatic capacity. We hypothesized that ticagrelor administered with a lower-than-standard, multiple-daily dose of aspirin establishes a beneficial hemostatic profile and so we investigated a novel regimen of aspirin 20 mg BD and ticagrelor 90 mg BD in patients treated for ACS.

## Methods

In the WILL lOWer dose aspirin be more effective in ACS? (WILLOW ACS) study, 20 patients with a history of recent ACS established on ticagrelor-based DAPT provided informed consent to participate in this study. To proceed to randomization, participants were required to meet the following inclusion criteria: male or female aged greater than 18 years; previous diagnosis of acute coronary syndrome greater than 30 days and less than 10 months before enrolment; and receiving DAPT with aspirin 75 mg OD and ticagrelor 90 mg BD. Participants were excluded if they met any of the following criteria: indication for DAPT other than ischaemic heart disease; percutaneous coronary intervention with drug-eluting or bare-metal stent(s) within 30 days prior to randomization; any history of stent implantation to the left main coronary artery; any history of stent thrombosis during DAPT; further planned coronary revascularization procedure; any planned surgery or other procedure that might require suspension or discontinuation of DAPT expected to occur within 3 months of randomization; prior intention by patient or physician to discontinue aspirin and/or ticagrelor within the study period; doses of aspirin and ticagrelor other than 75 mg OD and 90 mg BD, respectively; treatment or planned treatment with antiplatelet medication apart from aspirin or ticagrelor (eg. clopidogrel, prasugrel, dipyridamole or ticlopidine); currently receiving a diuretic agent (including loop, thiazide or potassium-sparing diuretics) as these may affect prostanoid assays; any ACS event within 30 days prior to randomization; most recent ACS event greater than 10 months prior to randomization; current or planned use of an oral anticoagulant (e.g. warfarin, dabigatran, rivaroxaban, apixaban), parenteral anticoagulant (e.g. unfractionated heparin, low-molecular-weight heparin, bivalirudin), glycoprotein IIb/IIIa inhibitor (e.g. abciximab, tirofiban) or fibrinolytic agent (e.g. tissue plasminogen activator); or requiring, or likely to require, treatment with a nonsteroidal anti-inflammatory drug, including intermittent/as required use; history of acute or chronic liver disease (e.g. cirrhosis), end-stage renal failure requiring dialysis, alcohol or drug abuse, defined as regular use of an illicit substance for recreational purposes or regular consumption of greater than 50 units (males) or 35 units (females) of alcohol per week in the last year; any comorbidity associated with life expectancy less than 1 year; or any other condition deemed by the investigator to affect hemostasis, coagulation, bleeding risk or ability to comply with the study protocol. To prevent interaction with ticagrelor treatment, those receiving a strong inhibitor of cytochrome P450 3A (e.g. ketoconazole, itraconazole, voriconazole, telithromycin, clarithromycin, nefazadone, ritonavir, saquinavir, nelfinavir, indinavir, atanazavir, or over 1 L daily of grapefruit juice), simvastatin or lovastatin at doses higher than 40 mg daily, a cytochrome P450 3A substrate with a narrow therapeutic index (e.g. cyclosporine or quinidine), or a strong inducer of cytochrome P450 3A (e.g. rifampin, rifabutin, phenytoin, carbamazepine, phenobarbital) were also excluded.

Females of child-bearing potential were similarly prevented from proceeding to randomization unless they had a negative pregnancy test at screening and were willing to use protocol-defined effective contraception for the duration of treatment with study medication.

### Study Design

**Figure 1.**
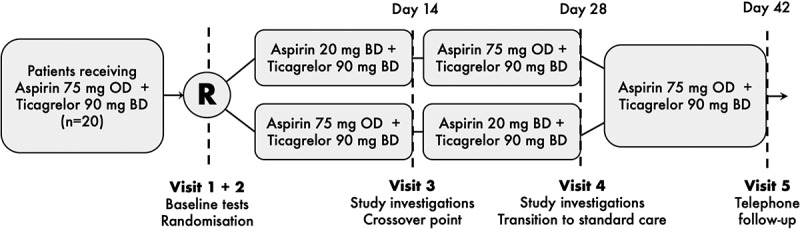
Overall design of the WILL lOWer dose aspirin be more effective in ACS? (WILLOW ACS) study. ACS, acute coronary syndrome; BD, twice daily; mg, milligrams; OD, once daily; R, point of randomization.

After collection of baseline demographic and clinical information, participants were randomized using opaque-sealed envelopes shuffled by an individual outside of the research team, in a 1:1 ratio, to one of two sequences of different regimens of soluble aspirin in an open-label crossover design (Figure 1):

1. Aspirin 20 mg BD (12-hourly, morning and evening) for 14 days *then* aspirin 75 mg OD (in the morning) for 14 days.
2. Aspirin 75 mg OD (in the morning) for 14 days *then* aspirin 20 mg BD (12-hourly, morning and evening) for 14 days.

All other usual medications, including ticagrelor 90 mg BD (12-hourly, morning and evening), were continued throughout the study in all participants.

Blood samples for serum thromboxane B_2_ (TXB_2_) and light transmittance aggregometry (LTA), urine samples for PGI_2_ metabolite and TX metabolite, and bleeding time measurement using a standard lancet method were obtained at the following time points:

1. After 14 days of study medication, pre-aspirin dose (platelet function and serum TXB_2_), representing trough effect, and 2 h post-dose (platelet function, serum TXB_2_, urinary prostanoids and bleeding time), representing peak effect.
2. After 28 days (end of second study medication period) pre-aspirin dose (platelet function and serum TXB_2_) and 2 h post-dose (platelet function, serum TXB_2_, urinary prostanoids and bleeding time).

Treatment periods of 14 days ensured that steady-state effects of aspirin were reached, whilst sampling 2 h after aspirin dose meant it could be confidently asserted that the time to maximal plasma concentration had elapsed, and so peak effects on platelet function, which persist beyond this point, were being observed [34,35].

Clinical outcomes were reviewed and adverse events recorded at 14 and 28 days. At the 28-day visit, patients with an indication for ongoing DAPT were transitioned to standard care with aspirin 75 mg OD and arrangements for ongoing supply of this medication were ensured. A telephone contact was made at 14 days after study medication discontinuation to ensure the successful transition to standard care and to record any adverse events for safety monitoring. Vital signs, physical examination findings and concurrent medications were recorded at each timepoint. On the last day of each medication period, participants were asked to take their ticagrelor on waking but delay aspirin until they attended for the study visit.

### Drug Supply and Accountability

To ensure accurate titration of aspirin doses, a fully soluble aspirin lysine preparation was used (Aspegic; Sanofi-Aventis, Machelen, Belgium). Each “100 mg” sachet contained 180 mg of aspirin lysine, including 100 mg of acetylsalicylic acid (aspirin). Participants were provided with tuition, illustrated written instructions and dosing equipment. They were asked to dissolve the whole of a 100 mg aspirin sachet in 100 mL of drinking water, measured in a graduated beaker (Sarstedt, Nümbrecht, Germany), stirring for 30 s to ensure full dissolution. To prepare 20 mg, they were asked to withdraw 20 mL of the solution using a marked syringe (Beckton-Dickinson, Franklin Lakes, New Jersey, USA) and ingest that amount, discarding the remainder. To dispense 75 mg they were asked to withdraw 25 mL and ingest the remainder. A new sachet was used for each dose to minimize drug hydrolysis once in solution.

Participants were provided with a medication diary to record times of aspirin and ticagrelor administration during the study, in order to assess compliance with study medication, which was also measured by counting returned aspirin sachets.

### Serum Thromboxane B_2_

Whole blood was collected into 5 mL serum separator tubes (Becton-Dickinson, New Jersey, USA) and immediately placed into a water bath, preheated to 37^o^C, for 30 min, as previously described [36,37]. Tubes were then centrifuged, serum was drawn off and stored at −80^o^C until analysis. Levels of the nonenzymatic metabolite of TXA_2_, TXB_2_, which in contrast to its precursor is highly stable in serum and therefore more suitable for measurement as a marker of platelet COX-1 activity [37], were estimated using a commercially available enzyme-linked immunosorbent assay (Cayman Chemical, Ann Arbor, USA). Samples were assessed twice and repeated if significant discrepancies were detected between the measurements. Interplate coefficient of variability (CV) for a standard mid-range concentration of TXB_2_ (62.5 pg/mL) was 14.3% in our laboratory, and mean intraplate CV was 9.5%.

### Light Transmittance Aggregometry

A broad assessment of platelet reactivity was made pre- and 2 h post-dose at the end of each treatment period using 6-min LTA with arachidonic acid (0.1, 0.3 and 1.0 mmol/L), collagen (1, 4 and 16 µg/mL) and adenosine diphosphate (20 µmol/L) as agonists (PAP-8 v2.0, Bio/Data Corporation, Horsham, PA, USA; stir speed 1200 rpm). Maximum aggregation response, adjusted for baseline aggregation, was recorded for each agonist. Samples were assessed in duplicate, taking the mean value for analysis, and repeated if a discrepancy of >10% was observed between the readings.

### Bleeding Time

Hemostasis was assessed using a standard lancet method (Haemolance Max Flow Plus, HTL-STREFA S.A., Poland). A calibrated sphygmomanometer cuff (Welch Allyn, Buckinghamshire, UK) was inflated around the upper arm to 40 mmHg and three incisions made on the volar aspect of the forearm. Each puncture site was checked for hemostasis every 30 s using filter paper (Whatman Grade 1, GE Healthcare, Little Chalfont, UK) until bleeding stopped or 30 min was reached. A mean of bleeding times was calculated. The method was validated in healthy volunteers receiving no medication (*n* = 5).

### Urinary Prostanoids

Midstream urine was collected around 2 h after the last study medication dose of each treatment period, centrifuged to remove any cellular material and then stored at −80^o^C prior to analysis. Urine PGI_2_ metabolite (2,3-dinor-6-keto prostaglandin F_1α_, PGI_2_ metabolite) was measured by gas chromatography-mass spectrometry, as previously described [38,39].

Urinary TX metabolite (11-dehydro-TXB_2_) and 8-iso prostaglandin F_2α_ (8-iso PGF_2α_), a marker of oxidant stress, were measured by previously validated immunoassays [40]. Urinary prostanoids were corrected for urinary creatinine levels, measured by the Department of Laboratory Medicine, Northern General Hospital, Sheffield, using a clinically accredited automated assay (Roche, Basel, Switzerland).

### Fibrin Clot Dynamics

Aspirin is known to affect fibrin clot dynamics [41,42]. To study the effects of the two aspirin regimens on clot formation and lysis, high-throughput turbidimetric analysis was performed as described and validated elsewhere [43–47]. Briefly, plasma samples were mixed with standard lysis and activation mixes to form acellular clots. Serial absorbance was measured using an automated plate reader until lysis was achieved. Variables recorded were maximum absorbance (a representation of fibrin clot turbidity), lag time (time from addition of clot activation mix to the start of clot formation) and lysis time (time taken for turbidity to drop by 50% from maximum as a measure of lysis potential).

### Safety Monitoring

Although primarily a pharmacodynamic study, we monitored safety throughout: the primary safety objective was to estimate the incidence of PLATO-defined major plus minor bleeding at 14 days in patients treated with ticagrelor and either one of the two aspirin dosing regimens [5]. Additionally, all adverse events (AEs) occurring between randomization and the telephone call 14 days after completion of study treatment were recorded. Serious adverse events (SAEs) were defined as any that resulted in death, was life-threatening, required hospitalization or prolongation of existing hospitalization, resulted in persistent disability or incapacity or consisted of a congenital abnormality or birth defect. Causality was assessed by the investigators. All AEs were followed up until resolved or stable.

### Statistical Analysis

The primary endpoint of the study was 2-h post-dose serum TXB_2_, compared within patients between the two dosing regimens by a paired t-test. Based on our previous data showing a mean serum TXB_2_ level of 2.9 ng/mL following ticagrelor 90 mg and aspirin 75 mg maintenance doses [36], we estimated that 20 patients entering the crossover design would have 80% power to detect a difference of 1.7 ng/mL in post-dose serum TXB_2_ between the regimens with alpha of 0.05. Based on previous data showing a mean circulating TXB_2_ level in non-aspirin-treated individuals (both healthy volunteers and patients with atherosclerotic disease) of around 45 ng/mL [15,48], we estimated that a difference of 1.7 ng/mL would represent a change of 3.8% in TXB_2_ inhibition.

Statistical analysis was performed using GraphPad PRISM version 7.03 (GraphPad Software Inc., La Jolla, CA, USA), using an alpha of 0.05 to determine significance. Continuous data are presented as mean ± standard deviation (SD). Where data were significantly skewed (as was the case for TXB_2_), log transformation was performed prior to analysis. Analysis was by intention to treat.

### Study Oversight

Approval to conduct the study was granted by the Sheffield National Research Ethics Service Committee, by the Medicines and Healthcare products Regulatory Authority and by the Health Research Authority prior to commencement of research activities, which were monitored by the Sheffield Teaching Hospitals National Health Service Foundation Trust Clinical Research Office. The study was registered with clinicaltrials.gov (NCT02741817) before commencing any research activity.

## Results

Twenty participants (16 males and 4 females) were randomized between July and December 2016, and all completed the study. Baseline characteristics are shown in Table I. Sampling times, compliance and physiological observations were similar between the two treatment regimens (Table II).

**Table I.** Baseline demographics of the 20 randomized patients.

Characteristic	–
Sex, male, *n* (%)	16 (80%)
Age, years, mean (SD)	64.3 (11.9)
Race	–
Caucasian	18 (90%)
Black	2 (10%)
Other	0 (0%)
Height, cm, mean (SD)	171.4 (10.4)
Weight, kg	–
Mean (SD)	83.6 (15.5)
Median (range)	86 (55–106)
BMI, kg/m^2^	–
Mean (SD)	28.3 (3.9)
Median (range)	28.6 (22.6–37.1)
Systolic BP, mmHg, mean (SD)	134.8 (20.1)
Diastolic BP, mmHg, mean (SD)	71.7 (11.7)
Pulse rate, bpm, mean (SD)	59.2 (5.6)
Diagnosis	–
STEMI	7 (35%)
NSTEMI	13 (65%)
Unstable angina	0 (0%)
Management of ACS	–
PCI	16 (80%)
CABG	0 (0%)
Conservative	4 (20%)
Smoking status	-
Current	2 (10%)
Past	11 (55%)
Never	7 (35%)
Diabetes mellitus	3 (15%)
Hypertension	11 (55%)
Hypercholesterolaemia	9 (45%)
Time from ACS event to randomization, days, mean (SD)	123.8 (82.8)
Concurrent medication	–
Beta-blocker	17 (85%)
ACE inhibitor/ARB	19 (95%)
Statin	19 (95%)
Regular nitrate	1 (5%)
Nicorandil	1 (5%)
Proton pump inhibitor	13 (65%)

ACE, angiotensin converting enzyme; ACS, acute coronary syndrome; ARB, angiotensin receptor blocker; CABG, coronary artery bypass grafting; NSTEMI, non-ST elevation myocardial infarction; PCI, percutaneous coronary intervention; STEMI, ST-elevation myocardial infarction; UA, unstable angina

**Table II.** Comparison of study medication period duration, sampling times and compliance between the 20 mg BD and 75 mg OD aspirin regimens. Values are shown as mean ± SD and *p* values were generated by paired t-tests.

–	20 mg BD	75 mg OD	*p* value
Time from aspirin dose to post-aspirin venepuncture (mins)	124.7 ± 18.2	123.1 ± 10.2	0.68
Time from last ticagrelor dose to pre-aspirin venepuncture (mins)	214.0 ± 208.5	184.9 ± 160.2	0.36
Time from aspirin dose to bleeding time measurement (mins)	122.2 ± 21.0	122.8 ± 19.6	0.88
Time from last aspirin dose to collection of urine sample (mins)	133.9 ± 24.7	136.2 ± 24.6	0.76
Time of aspirin dose on day of sampling from previous dose (hrs)	13.7 ± 2.4	26.2 ± 1.8	NA
Compliance with aspirin therapy (% of doses taken)	99.9 ± 0.7	100.0 ± 0.0	0.33
Compliance with ticagrelor therapy (% of doses taken)	100.0 ± 0.0	99.7 ± 1.3	0.33
Time on study medication (days)	14.95 ± 2.0	14.80 ± 1.47	0.55
Systolic BP (mmHg)	127.3 ± 12.9	128.8 ± 15.5	0.67
Diastolic BP (mmHg)	72.2 ± 10.2	71.1 ± 10.2	0.53
Heart rate (bpm)	60.7 ± 6.0	62.3 ± 10.3	0.49

BD, twice daily; BP, blood pressure; bpm, beats per minute; hrs, hours; mmHg, millimeters of mercury; Mins, minutes; NA, not applicable; OD, once daily

### Serum TXB_2_

**Figure 2.**
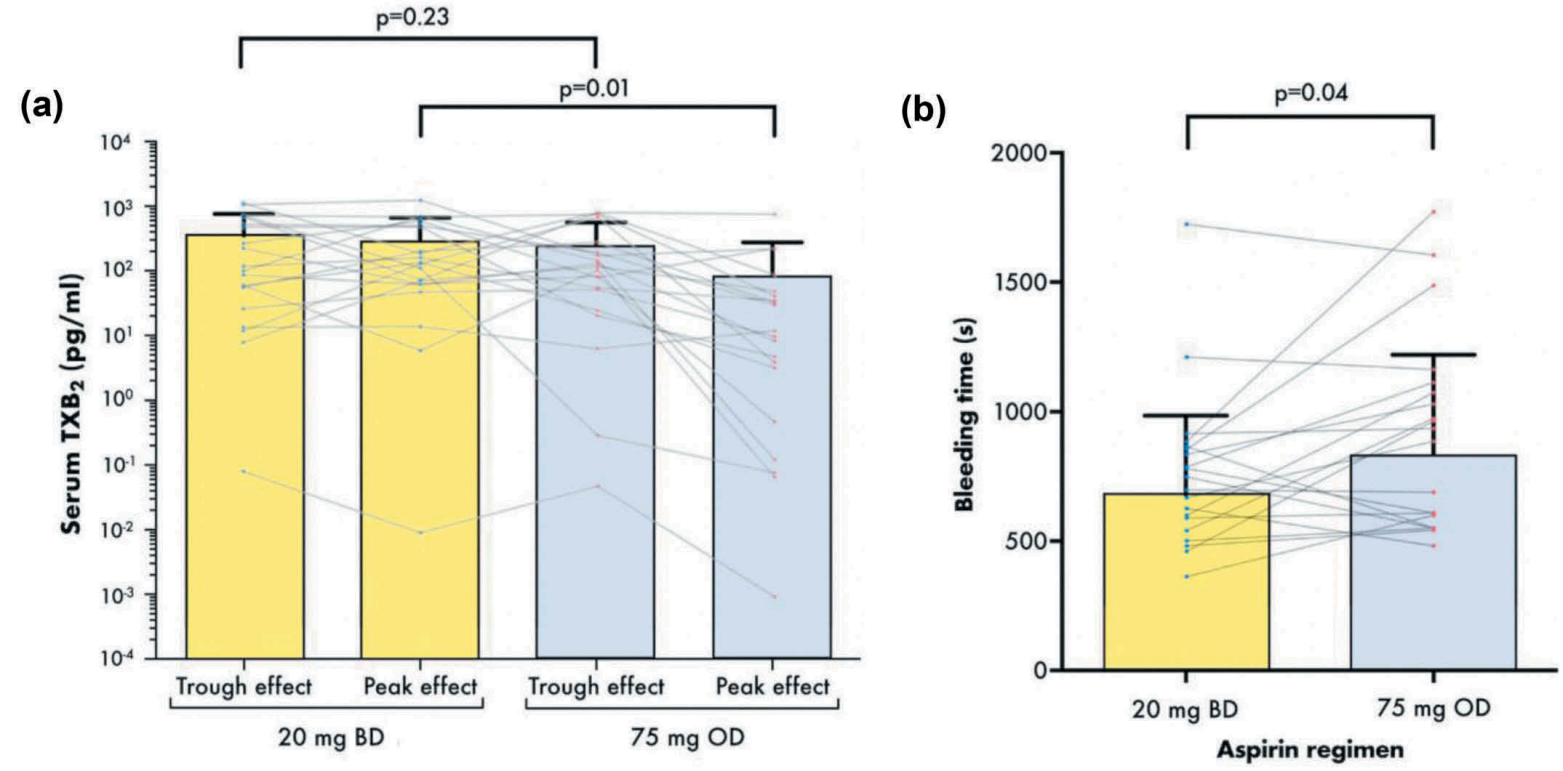
Serum TXB_2_ (a) and bleeding time (b) in ACS patients receiving two regimens of aspirin and ticagrelor in the WILLOW ACS study. Bars represent mean + SD. Dots and lines represent paired values for the individual participants. *p* values shown were generated by paired t-tests between the groups (see text and tables for further details). BD, twice-daily; OD, once-daily s, seconds; sTXB2, serum thromboxane B2. the groups (see text and tables for further details). Scale on the *y*-axis in Figure 2(a) is logarithmic. BD, twice-daily; OD, once-daily s, seconds; TXB_2_, thromboxane B_2_.

At steady state, mean (± SD) serum TXB_2_ levels 2 h post-dose were significantly greater when receiving 20 mg BD when compared to 75 mg OD (3.03 ± 3.64 ng/mL vs. 0.83 ± 1.93 ng/mL, *p* = 0.018) (Table III, Figure 2(a)) and there was no significant difference between the regimens at pre-dose, which corresponds to the trough effect (3.51 ± 4.07 ng/mL vs. 2.48 ± 3.14 ng/mL, *p* = 0.23) (Table III, Figure 2(a)). There was no evidence of a carryover effect between the dosing regimens: no significant differences in pre- or post-dose TXB_2_ measurements were observed between the participants who received 20 mg BD in the first period vs. the second (pre-dose *p* = 0.768, 2 h post-dose *p* = 0.774, data not shown) and between those who received 75 mg OD in the first vs. the second (pre-dose *p* = 0.907, post-dose *p* = 0.914, data not shown).

**Table III.** Results of analyses of pharmacodynamic endpoints during each aspirin dosing regimen at steady state. Values shown are mean ± SD. *p* values were generated from paired t-tests.

–		20 mg BD	75 mg OD	*p* value
**PRE-DOSE (TROUGH EFFECT)**				
Serum TXB_2_ (ng/mL)		3.51 ± 4.07	2.48 ± 3.14	0.23
Light transmittance aggregometry—maximum aggregation (%)				
*Agonist*	*[Agonist]*	–		
AA (mmol/L)	0.1	2.5 ± 1.8	2.4 ± 1.6	0.62
	0.3	2.2 ± 1.3	1.7 ± 1.5	0.10
	1	8.7 ± 14.2	6.6 ± 5.3	0.41
Collagen (µg/mL)	1	26.3 ± 20.3	15.5 ± 9.4	0.03
	4	57.0 ± 15.7	51.0 ± 18.1	0.16
	16	72.1 ± 11.8	73.5 ± 9.1	0.60
ADP (µmol/L)	20	44.6 ± 11.7	41.5 ± 12.0	0.21
**POST-DOSE (PEAK EFFECT)**				
Serum TXB_2_ (ng/mL)		3.03 ± 3.64	0.83 ± 1.93	0.018
Urinary TxM (pg/mg creatinine)		430.0 ± 269.7	371.5 ± 176.6	0.17
Urinary PGI-M (pg/mg creatinine)		109.9 ± 143.3	86.68 ± 54.58	0.41
Urinary 8-iso-PGF_2α_ (pg/mg creatinine)		2713 ± 1534	2834 ± 1945	0.77
Bleeding time (s)		679.5 ± 305.5	833.9 ± 385.7	0.04
Light transmittance aggregometry—maximum aggregation (%)				
*Agonist*	*[Agonist]*	–		
AA (mmol/L)	0.1	2.3 ± 1.5	2.6 ± 1.5	0.50
	0.3	2.1 ± 1.6	1.7 ± 0.9	0.23
	1	8.5 ± 14.3	5.1 ± 3.6	0.24
Collagen (µg/mL)	1	16.5 ± 15.6	9.0 ± 5.7	0.03
	4	44.6 ± 23.3	29.6 ± 14.9	0.007
	16	65.6 ± 12.0	58.4 ± 13.7	0.11
ADP (µmol/L)	20	40.9 ± 15.5	40.6 ± 12.1	0.86

AA, arachidonic acid; ADP, adenosine diphosphate; BD, twice-daily; PGI-M, prostacyclin metabolite; OD, once-daily; s, seconds; TXB_2_, thromboxane B_2_

### Bleeding Time

Bleeding time measured 2 h post-dose in each treatment period was significantly shorter when receiving aspirin 20 mg BD compared to 75 mg OD (679.5 ± 305.5 vs. 833 ± 385.7 s, *p* = 0.04), a mean reduction of 154.5 s (18.5%) (Table III, Figure 2(b)). For comparison, mean ± SD bleeding time in five healthy drug-free volunteers was 342 ± 72 s, with a mean intrasubject variation of 24 s (7%).

### Light Transmittance Aggregometry

**Figure 3.**
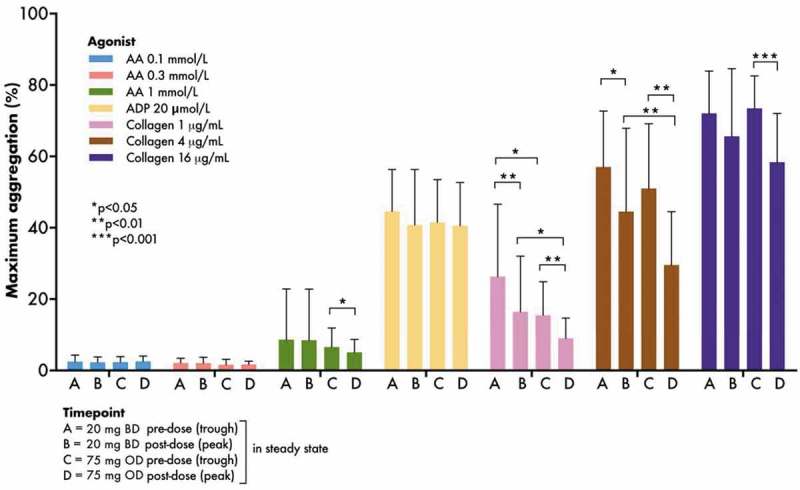
Maximum platelet aggregation responses to arachidonic acid, adenosine diphosphate and collagen assessed by light transmittance aggregometry pre- and post-aspirin dose at the end of each treatment period in the WILLOW ACS study. Bars indicate mean + SD. *p* values were generated using prespecified paired t-tests. AA, arachidonic acid; ADP, adenosine diphosphate; BD, twice-daily; OD, once-daily.

There were no significant differences in arachidonic acid- or adenosine diphosphate-induced maximum aggregation responses between the regimens (Table III, Figure 3).

Two-hours post-dose maximum aggregation responses to collagen 1 and 4 µg/mL (but not 16 µg/mL) were greater when receiving aspirin 20 mg BD compared to 75 mg OD (mean ± SD: 20 mg BD 16.5 ± 15.6% vs. 75 mg OD 9.0 ± 5.7%, *p* = 0.03; and 20 mg BD 44.6 ± 23.3 vs. 75 mg OD 29.6 ± 14.9%, *p* = 0.007 for collagen 1 and 4 µg/mL, respectively). Pre-dose maximum aggregation to collagen 1 µg/mL (26.3 ± 20.3 vs. 15.5 ± 9.4%, *p* = 0.03), but not 4 or 16 µg/mL, was greater when receiving aspirin 20 mg BD (Table III, Figure 3). Given the universally low, though expected, responses to arachidonic acid, the efficacy of the agonist was tested in drug-free platelet-rich plasma from healthy volunteers (*n* = 8): mean (± SD) maximum aggregation in response to stimulation with 1 mmol/L arachidonic acid was 79.1 ± 7.4%.

### Urinary Prostanoids

**Figure 4.**
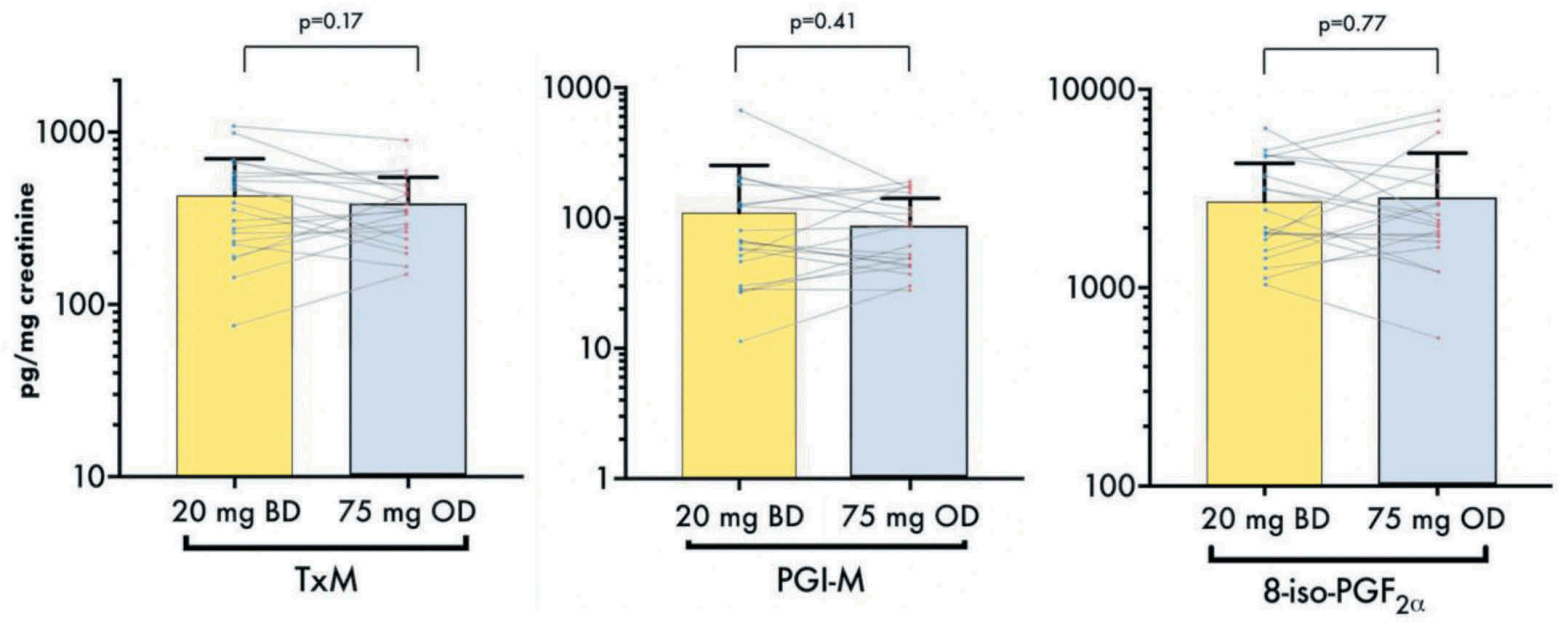
Urinary levels of TX metabolite, PGI_2_ metabolite 8-iso-PGF_2α_ measured at the end of each treatment period. Bars represent mean + SD. Dots and lines represent paired values for the individual participants. *p* values shown were generated by paired t-tests between the groups. Scale on the *y*-axis is logarithmic. 8-iso-PGF_2α_, 8-iso prostaglandin F_2α_; BD, twice- daily; PGI-M, prostacyclin metabolite; OD, once-daily; TxM, thromboxane metabolite.

Urinary TX metabolite, which represents COX-1 activity over a broader time range than serum TXB_2_, showed no significant difference comparing BD vs. OD regimens (430.0 ± 269.7 vs. 371.5 ± 176.6 pg/mg creatinine, *p* = 0.17 for paired comparison). Urine PGI-M and 8-iso-PGF_2α_ levels did not significantly differ between the two groups (Table III, Figure 4).

### Fibrin Clot Parameters

Given the importance of fibrin network lysis in cardiovascular outcome following acute coronary syndrome [47], and the effects of aspirin on the fibrin clot [41], we studied potential differences in a number of fibrin clot properties comparing the two different dosing regimens of aspirin. We found no significant differences in lag time (a measure of clotting tendency), clot maximum absorbance (a measure of clot density and fiber thickness) or lysis time (a measure of resistance of clot to breakdown) between the regimens pre- or post-aspirin dosing in the two groups studied (supplementary material).

### Safety

There were no PLATO-defined major or minor bleeding events and no SAEs occurred during the study, including any thrombotic events. Two participants suffered trivial bleeding events during the aspirin 75 mg OD period, compared to none whilst receiving aspirin 20 mg BD (Supplementary Table I). No participant stopped study medication prematurely.

### Effect of Weight and Body Mass Index

Study participants had a median enrolment weight of 86 kg (range 55–106) and body mass index (BMI) of 28.6 kg/m^2^ (22.6–37.1). There were no significant correlations between body weight or BMI and TX metabolite or PGI_2_ metabolite when receiving either the novel or standard regimen (Supplementary Figure 1), suggesting the efficacy of the novel regimen was maintained across the range of weight and BMI.

## Discussion

DAPT with aspirin and ticagrelor represents a standard maintenance antithrombotic therapy that is recommended as first-line treatment following ACS [3,4,49]. Typically given for at least 1 year, continuation for longer remains an effective strategy for preventing MACE in high-risk patients but comes at the price of increased bleeding [6]. Even though the overall balance of mortality risks appears to favor use of longer-term ticagrelor-based DAPT in high-risk patients [50–52], clinicians and patients alike may be reluctant to extend DAPT therapy due to the bleeding risk. It has been proposed that aspirin can safely be stopped in ticagrelor-treated patients with a history of percutaneous coronary intervention [53], an approach tested in the recently reported GLOBAL LEADERS study, which showed that 1 month of DAPT followed by 23 months of ticagrelor monotherapy was not superior to 12 months of DAPT followed by 12 months of aspirin monotherapy, although this was limited by heterogeneity of P2Y_12_ receptor antagonists in the control group [54]. Even if ticagrelor monotherapy is effective in preventing stent thrombosis, there are likely to remain a significant group of patients at high risk of ongoing native plaque rupture events in whom DAPT is needed to optimize protection against future MACE.

**Figure 5.**
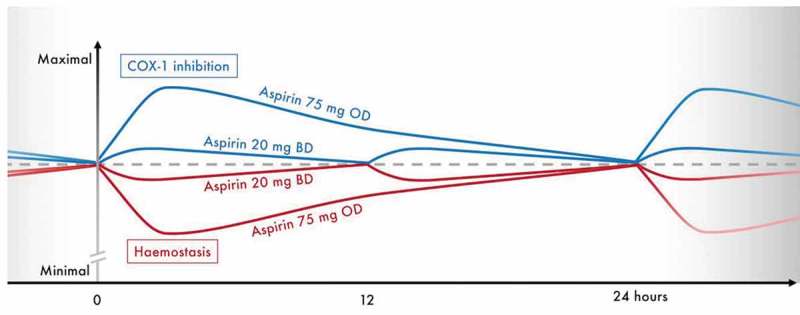
Conceptual figure summarizing the anticipated profiles of COX-1 inhibition and hemostasis provided by maintenance aspirin doses of 75 mg OD or 20 mg BD in combination with ticagrelor 90 mg BD, during steady-state. Figure for illustrative purposes only, scale on the *y*-axis is arbitrary. Dashed line represents effects on COX-1 and hemostasis at steady-state trough levels. BD, twice-daily; COX-1, cyclo-oxygenase 1; mg, milligrams; OD, once-daily.

Strategies maintaining the combined antithrombotic effect of DAPT, whilst reducing bleeding tendency, therefore have the potential to improve overall clinical outcomes. We have shown that a novel regimen of very-low-dose BD aspirin given to ticagrelor-treated ACS patients broadly maintains the inhibitory effects of aspirin on TXA_2_ synthesis and arachidonic acid-induced platelet activation, but reduces peak inhibition and is associated with a significant reduction in bleeding time (Figure 5). It remains to be determined whether this will translate into a reduction in clinical bleeding events, but similar doses have previously been shown to have clinical antithrombotic efficacy. The European Stroke Prevention Study 2 investigated a similar very-low-dose, BD aspirin regimen (25 mg BD) alone or in combination with another antiplatelet drug (dipyridamole) in 6602 stroke patients, showing a significant benefit of BD aspirin, alone or in combination, vs. placebo in preventing recurrent cerebrovascular events [55]. Giving aspirin BD may also improve symmetry of DAPT effect when given with ticagrelor, which is also given BD, and may simplify drug intake, including the possibility of developing a combination tablet. This might help to address the under-recognized issue of treatment compliance that can limit the efficacy of treatment strategies in coronary artery disease patients [56], and can be improved by reducing the number of tablets they receive [57].

The use of very-low-dose twice-daily aspirin in this setting is only considered feasible when given in combination with ticagrelor which, in contrast to older drugs, provides potent and reliable P2Y_12_ inhibition [58]. P2Y_12_ inhibitors reduce AA-induced platelet aggregation independently of aspirin [59], meaning there may be a rationale for reducing the intensity of aspirin therapy. However, the results of our study also illustrate the fact that aspirin continues to provide additional antiplatelet effects even in the presence of potent P2Y_12_ inhibition, given that we saw differences in TX-related biomarkers and platelet function between the two dosing regimens at 2 h post-dose. This is consistent with previous studies of the relationship between aspirin and P2Y_12_ receptor inhibition or deficiency [60]. Hence, there is justification for an approach that continues to include aspirin but seeks to reduce its intensity to improve hemostasis whilst maintaining adequate levels of platelet inhibition.

In pre-dose samples, we saw similar levels of overall platelet inhibition with the two regimens, but it is possible there may be differences at the platelet level in the pattern of inhibition between the two regimens at this timepoint given that some newly formed platelets will be more rapidly exposed to aspirin with the BD regimen. Even a small number of uninhibited platelets can form the basis for thrombosis [61]. However, the clinical efficacy and safety of a BD aspirin regimen remains to be explored.

The study was limited by a small sample size and therefore was unable to determine whether the very-low-dose aspirin regimen significantly improves *in vivo* prostacyclin biosynthesis so this will require assessment in a larger study. The goal of the current study was to provide reassurance from a pharmacodynamic study that such a larger study is appropriate. Similarly, we only compared one novel regimen with standard therapy, rather than including multiple permutations; however, our results suggest that aspirin 20 mg BD and ticagrelor 90 mg BD achieve our goal of reducing peak-trough variation in effect and improving hemostasis.

We saw no significant correlation between weight or BMI and COX-1 inhibition in the present study (supplementary material) but lacked sufficient numbers of obese patients, with or without diabetes, to determine levels of COX-1 inhibition in these individuals who tend to have diminished response to low-dose aspirin [62]. Consequently, further pharmacodynamic studies focussing on obese or diabetes patients are required to understand how aspirin regimens may potentially be modified in these individuals when combined with ticagrelor.

In conclusion, aspirin dose modification represents a novel and feasible strategy to be investigated for optimizing the balance of antithrombotic benefits and bleeding-related risks in ticagrelor-treated ACS patients, and demands further study to determine whether this translates into improvements in net clinical outcomes.
