# Supplementary material for: Very-low-dose twice-daily aspirin maintains platelet inhibition and improves haemostasis during dual-antiplatelet therapy for acute coronary syndrome
Source: Platelets. 2019 Feb 13;30(2):148–57. doi: 10.1080/09537104.2019.1572880 (PMC6425913; doi:10.1080/09537104.2019.1572880)
Supplement: Supplemental Material [file IPLT_A_1572880_SM0243.docx]

**SUPPORTING INFORMATION**

**Supporting table 1.** Adverse events occurring during each treatment regimen

|  | Aspirin regimen | | Severity | Serious? |
| --- | --- | --- | --- | --- |
|  | 20 mg BD (n=20) | 75 mg OD  (n=20) |  |  |
| **ADVERSE EVENTS** | | | | |
| *Possibly related to study medication* | | | | |
| Epistaxis (not requiring medical attention) | 0 | 1 | Mild | No |
| Spontaneous cutaneous bruising | 0 | 1 | Mild | No |
|  | | |  |  |
| *Unlikely to be related to study medication* | | | | |
| Non-cardiac chest pain* | 1 | 0 | Mild | No |
| Pedal oedema | 0 | 1 | Mild | No |
| Lower respiratory tract infection | 0 | 1 | Moderate | No |

*In addition, 1 participant developed non-cardiac chest pain (secondary to trauma) after transition back to standard-of-care aspirin 75 mg OD, which was noted at the telephone follow-up visit.

BD, twice-daily; mg, milligrams; OD, once-daily.

B

A


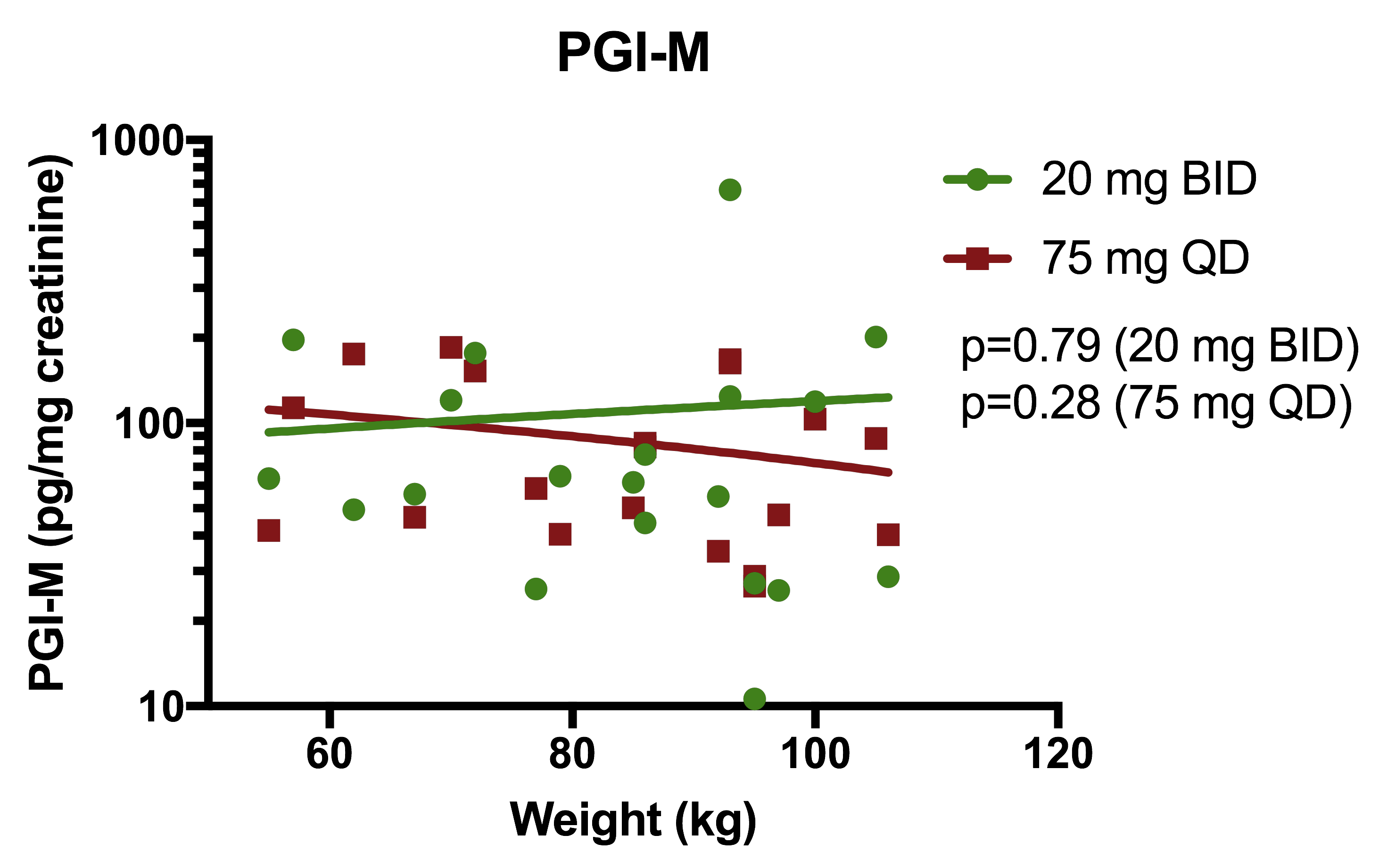

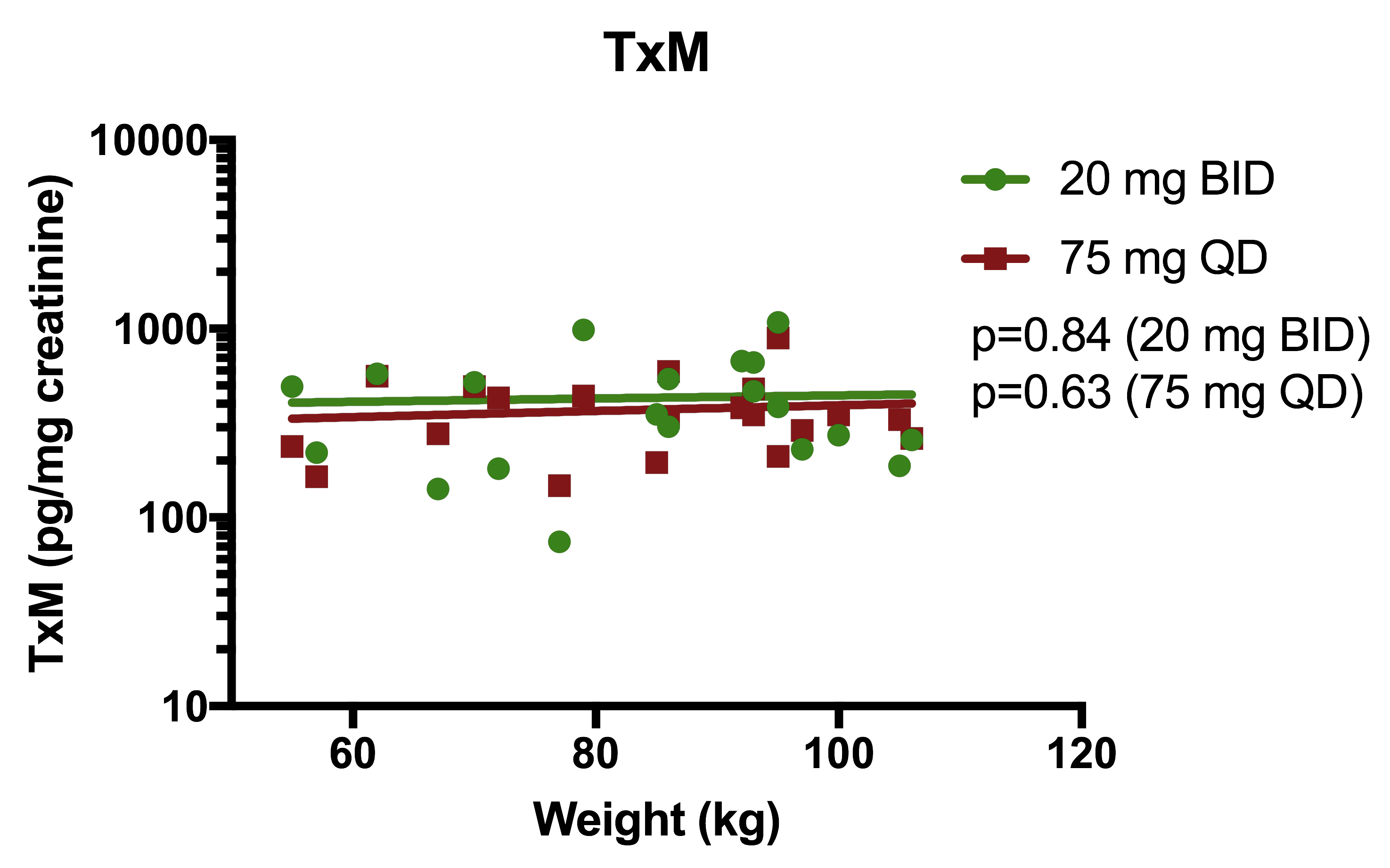


20 mg BD

75 mg OD

20 mg BD

75 mg OD

20 mg BD

75 mg OD

20 mg BD

75 mg OD

(20 mg BD)

(75 mg OD)

(20 mg BD)

(75 mg OD)


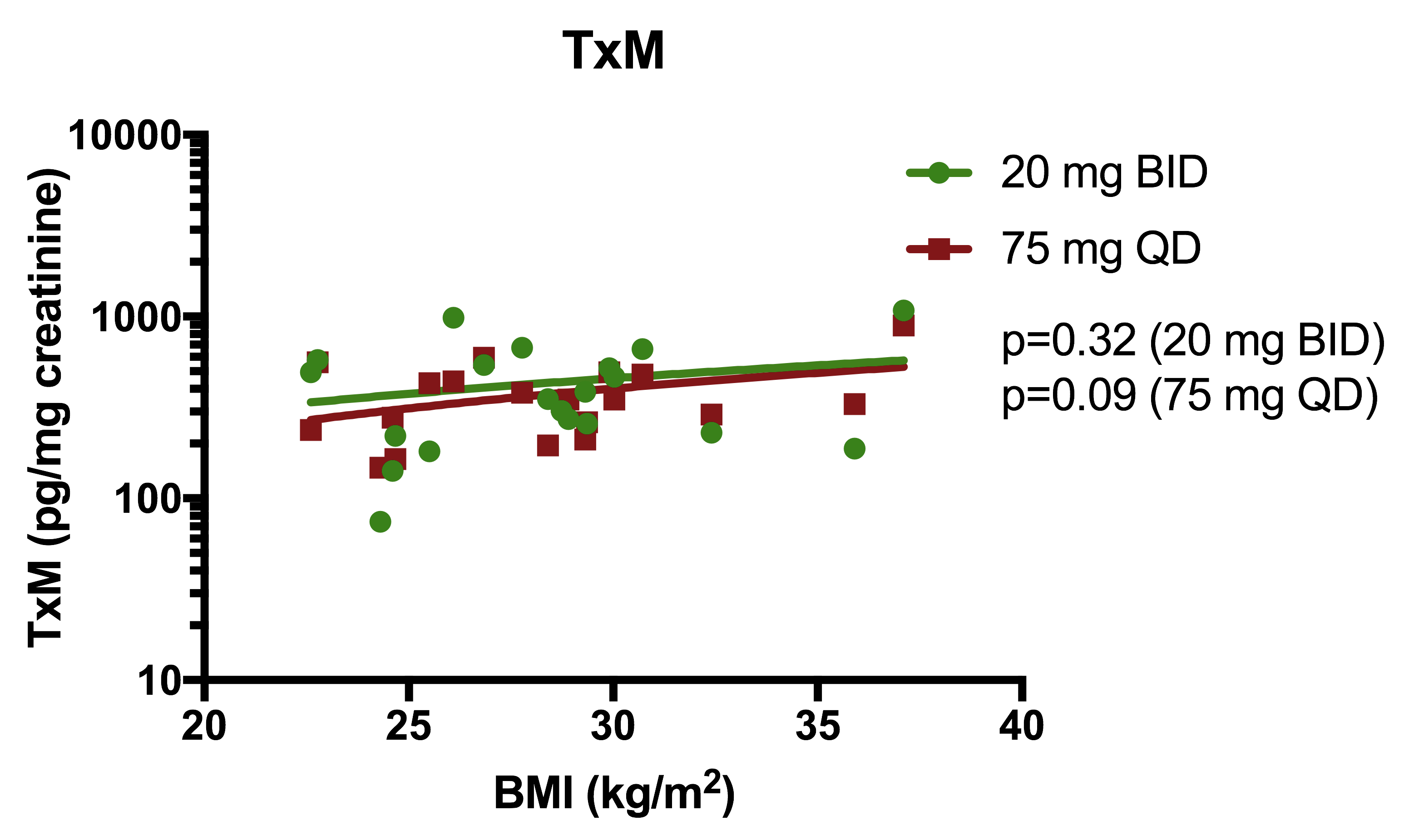

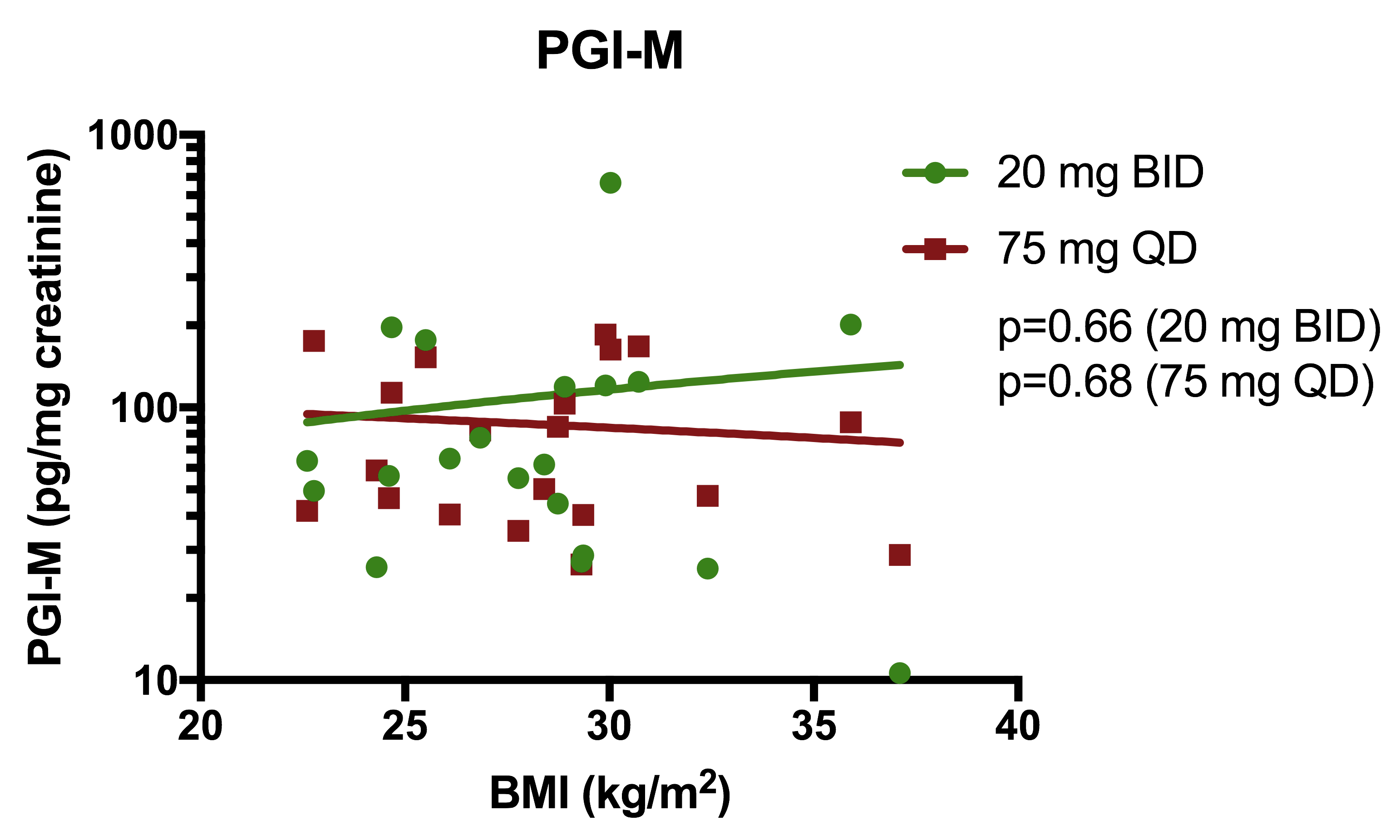


C

D

20 mg BD

75 mg OD

20 mg BD

75 mg OD

20 mg BD

75 mg OD

20 mg BD

75 mg OD

(20 mg BD)

(75 mg OD)

(20 mg BD)

(75 mg OD)

**Supporting figure 1** Correlation between body weight and urine TX metabolite (TxM) (panel A) or PGI_2_ metabolite (PGI-M) (panel B); and body mass index (BMI) and TxM (panel C) or PGI-M (panel D) in ticagrelor-treated patients receiving aspirin 20 mg BD or 75 mg OD, assessed by linear regression.

**Supporting table 2** Dynamics of fibrin clot formation and lysis in ticagrelor-treated ACS patients receiving aspirin 20 mg BD or 75 mg OD, assessed by turbidimetry. Values represent mean ± SD. P values were generated using paired t-tests. AU, absorbance units; s, seconds.

|  | **20 mg BD** | **75 mg OD** | **p value** |
| --- | --- | --- | --- |
| **PRE-DOSE** | | | |
| Maximum turbidity (AU) | 0.410 ± 0.089 | 0.397 ± 0.087 | 0.51 |
| Lag time (s) | 475.5 ± 86.8 | 494.7 ± 107.6 | 0.70 |
| Lysis time (s) | 759.9 ± 334.3 | 745.3 ± 470.0 | 0.97 |
| **POST-DOSE** | | | |
| Maximum turbidity (AU) | 0.394 ± 0.089 | 0.411 ± 0.111 | 0.52 |
| Lag time (s) | 496.3 ± 107.5 | 500.8 ± 78.94 | 0.87 |
| Lysis time (s) | 680.7 ± 313.1 | 618.6 ± 151.4 | 0.631 |

C

B

A

**Supporting figure 2** Maximum turbidity (A), lysis time (B) and lag time (C) of fibrin clot in ticagrelor-treated ACS patients receiving aspirin 20 mg BD or 75 mg OD, assessed by turbidimetry. Bars represent mean ± SD. No comparisons between 20 mg BD (pre-dose) and 75 mg (pre-dose), or between 20 mg BD (post-dose) and 75 mg OD (post-dose) showed a significant difference. AU, absorbance units; s, seconds.
